# Supplementary material for: Development of a Japanese version of the Psychological Ownership Scale
Source: PeerJ. 2022 Mar 24;10:e13063. doi: 10.7717/peerj.13063 (PMC8957756; doi:10.7717/peerj.13063)
Supplement: Supplemental Information 1 [file peerj-10-13063-s001.docx]

**Estimation of necessary sample size and exclusion criteria**

**Study 1**

Survey 1 participants were recruited using a crowdsourcing service. Walasek, Matthews, and Rakow (2015) performed principal component analysis on data from 732 participants; thus, we considered the analysis of an equivalent sample size to be necessary for our survey as well. We selected 800 as our required sample size because of the difficulty in recruiting an odd number of participants through the Yahoo! crowdsourcing system. To ensure sample independence, we performed exploratory factor analysis and confirmatory factor analysis using separate data (Iwasa et al., 2016). The necessary sample size for these factor analyses was 360 participants (20 × number of items × 2 data; Hair et al., 1995). Considering the need to exclude participants showing undesirable response behaviors, such as minimizing effort (e.g., Oppenheimer, Meyvis, & Davidenko, 2009) from analysis, we selected a maximum sample size of 900 participants. Excluding satisficers through preliminary analysis after our initial recruitment revealed that we were below our required sample size, and hence we conducted supplemental recruitment.

A total of 1,212 participants completed the survey. We excluded those who selected any answer other than “neither” for the question “Do you like the font of these instructions?” (i.e., IMC), and those who gave incorrect responses to the numerical calculation question (i.e., ACQ). Further, we excluded participants who responded with 0 yen for WTP or market price because these values were to be used as denominators for division when conducting the analysis. Ultimately, data from 376 participants were excluded, and data from 836 participants were analyzed.

Survey 2 participants were also recruited using a crowdsourcing service. Power analysis using G*Power (Faul et al., 2007) resulted in an estimated sample size of 995 participants (multiple regression analysis; small effect size, Cohen’s *f*^2^ = 0.02, α = .05, 1-β = .95, five explanatory variables). In addition to the exclusion criteria from Survey 1 (31% exclusion rate in Survey 1), Survey 2 followed Walasek et al. (2015) to exclude participants who responded with a WTA of 10 times the market price or higher. Thus, we aimed to recruit 1,600 participants. A total of 1,555 participants completed the survey, of which data from 714 were excluded, resulting in the analysis of data from 841 participants.

Survey 3 participants were recruited from university students. Power analysis using G*Power resulted in an estimated sample size of 138 (correlation analysis; two-tailed, medium effect size, *r* = .03，α = .05, 1-β = .95). Assuming that participants who did not complete the second survey required to confirm test-retest reliability would be excluded in addition to the 31% exclusion rate from Survey 1, a class with 247 enrollees was recruited. The survey was administered twice, with a four-week interval. There were 205 participants in administration one and 194 in administration two. Of the 127 participants who provided complete answers at both administrations and were not excluded as satisficers, an additional 22 were excluded for thinking of different objects at administration one than at administration two. Ultimately, data from 105 participants were analyzed.

**Study 2**

We assumed that we would compare the means of two different groups. Power analysis using G*Power resulted in an estimated sample size of 105 participants for each group (total 210 participants) (two-tailed, Cohen’s d = 0.5, α = .05, 1-β = .95, Allocation ratio = 1), which was selected as the required sample size. As the experiment was conducted as part of a class with 253 college students, the maximum sample size was 253. Data from 252 students were analyzed, excluding one student with missing responses to the POS-J questions.

**References**

Faul F, Erdfelder E, Lang AG, Buchner A. 2007. G*Power 3: A flexible statistical power analysis program for the social, behavioral, and biomedical sciences. *Behavior Research Methods* 39:175–191.

Hair JF, Jr., Anderson RE, Tatham RL, Black WC. 1995. Multivariate data analysis (4th ed.). Saddle River, NJ: Prentice Hall.

Iwasa K, Tanaka T, Yamada Y. 2016. Factor structure, reliability, and validity of the Japanese version of the disgust propensity and sensitivity scale-revised. *PLOS ONE* 11:e0164630.

Oppenheimer DM, Meyvis T, Davidenko N. 2009. Instructional manipulation checks: Detecting satisficing to increase statistical power. *Journal of Experimental Social Psychology* 45:867–872.

Walasek L, Matthews WJ, Rakow T. 2015. The need to belong and the value of belongings: Does ostracism change the subjective value of personal possessions? *Journal of Behavioral and Experimental Economics* 58:195–204.
