# Supplementary material for: Development of a Japanese version of the Psychological Ownership Scale
Source: PeerJ. 2022 Mar 24;10:e13063. doi: 10.7717/peerj.13063 (PMC8957756; doi:10.7717/peerj.13063)
Supplement: Supplemental Information 2 [file peerj-10-13063-s002.pdf]

# Supplemental Tables

**Table 1**

**Japanese version of the Psychological Ownership Scale**

| Items |
| --- |
| English (Walasek, Matthews & Rakow, 2015) |
| Possession-self link |
| That is part of who I am. |
| I derive some of my identity from that. |
| That is central to my identity. |
| That helps me narrow the gap between what I am and what I try to be. |
| That helps me to achieve the identity I wish to have. |
| That and I have a lot in common. |
| Feeling of ownership |
| I feel like I own that. |
| I feel like that is my possession. |
| I feel a very high degree of personal ownership of that. |
| Japanese |
| 所有物・自己関連性因子 |
| それは，私という人間の一部である。 |
| 私は，自分のアイデンティティの一部をそれから得ている。 |
| それは，自分のアイデンティティの中心である。 |
| それは，実際の自分となりたい自分とのギャップを埋める助けをしてくれる。 |
| それは，私が望むようなアイデンティティを獲得する助けをしてくれる。 |
| それと私には，たくさんの共通点がある。 |
| 所有感覚因子 |
| 私は，それを所有しているような気がする。 |
| それは，私の所有物であるように感じる。 |
| 私は，それに対して高い所有権を有しているように感じる。 |

Note) This scale is intended for use with owned objects.

**Table 2**

**Self-extension tendency scale**

| Items |
| --- |
| English（Ferraro, Escalas, & Bettman, 2011) |
| I have a special bond with my favorite possessions. |
| I consider my favorite possessions to be a part of myself. |
| I often feel a personal connection between my special possessions and me. |
| Part of me is defined by the special possessions in my life. |
| I feel as if I have a close personal connection with the possessions I most prefer. |
| I can identify with important possessions in my life. |
| There are links between my special possessions and how I view myself. |
| My favorite possessions are an important indication of who I am. |
| Japanese |
| 私は，自分のお気に入りの持ち物と特別なつながりがある。 |
| 私は，自分のお気に入りの持ち物を自分の一部だと思っている。 |
| 私はよく自分にとって特別な持ち物と自分との間に，個人的なつながりがあると感じる。 |
| 私の一部は，人生において特別な持ち物によって定義される。 |
| 私は，自分が最も好きな持ち物と親密で個人的なつながりがあるかのように感じる。 |
| 私は，人生において重要な持ち物を見極めることができる。 |
| 自分にとって特別な持ち物と，自分自身をどう見るかということとの間には，関連がある。 |
| 自分のお気に入りの持ち物は，私という人間を示すのに重要だ。 |

**Table 3**

**Factor loadings for confirmatory factor analysis**

| Items | Factor loadings |
| --- | --- |
| Possession-self link | |
| That is part of who I am. | .70 |
| I derive some of my identity from that. | .82 |
| That is central to my identity. | .79 |
| That helps me narrow the gap between what I am and what I try to be. | .63 |
| That helps me to achieve the identity I wish to have. | .80 |
| That and I have a lot in common. | .59 |
| Feeling of ownership | |
| I feel like I own that. | .85 |
| I feel like that is my possession. | .85 |
| I feel a very high degree of personal ownership of that. | .75 |

**Table 4**

**Japanese version of a Psychological Ownership Scale (No-legal-ownership Version)**

| Items |
|---|
| English |
| Possession-self link |
| I feel as if that is part of who I am. |
| I feel as if I derive some of my identity from that. |
| I feel as if that is central to my identity. |
| I feel like that helps me narrow the gap between what I am and what I try to be. |
| I feel like that helps me achieve the identity I wish to have. |
| I feel like that and I have a lot in common. |
| Feeling of ownership |
| I feel as if I own that. |
| I feel as if that is my possession. |
| I feel as if I have a very high degree of personal ownership of that. |
| Japanese |
| 所有物・自己関連性因子 |
| **まるで**，それは私という人間の一部である**かのように感じる**。 |
| **まるで**，私は自分のアイデンティティの一部をそれから得ている**かのように感じる**。 |
| **まるで**，それは自分のアイデンティティの中心である**かのように感じる**。 |
| それは，実際の自分となりたい自分とのギャップを埋める助けをしてくれる**ような気がする**。 |
| それは，私が望むようなアイデンティティを獲得する助けをしてくれる**ような気がする**。 |
| それと私には，たくさんの共通点がある**ような気がする**。 |
| 所有感覚因子 |
| **まるで**，私はそれを所有している**かのように感じる**。 |
| **まるで**，それは私の所有物である**かのように感じる**。 |
| **まるで**，私はそれに対して高い所有権を有している**かのように感じる**。 |

Note) This scale is intended for use with non-owned items.
