# Supplementary material for: Development of a Japanese version of the Psychological Ownership Scale
Source: PeerJ. 2022 Mar 24;10:e13063. doi: 10.7717/peerj.13063 (PMC8957756; doi:10.7717/peerj.13063)
Supplement: Supplemental Information 4 [file peerj-10-13063-s004.pdf]

Test Instrument in Japanese

POS-J

1. それは，私という人間の一部である。
2. 私は，自分のアイデンティティの一部をそれから得ている。
3. それは，自分のアイデンティティの中心である。
4. それは，実際の自分となりたい自分とのギャップを埋める助けをしてくれる。
5. それは，私が望むようなアイデンティティを獲得する助けをしてくれる。
6. それと私には，たくさんの共通点がある。
7. 私は，それを所有しているような気がする。
8. それは，私の所有物であるように感じる。
9. 私は，それに対して高い所有権を有しているように感じる。

POS-J (No-legal-ownership version)

1. まるで，それは私という人間の一部であるかのように感じる。
2. まるで，私は自分のアイデンティティの一部をそれから得ているかのように感じる
3. まるで，それは自分のアイデンティティの中心であるかのように感じる。
4. それは，実際の自分となりたい自分とのギャップを埋める助けをしてくれるような気がする。
5. それは，私が望むようなアイデンティティを獲得する助けをしてくれるような気がする。
6. それと私には，たくさんの共通点があるような気がする。
7. まるで，私はそれを所有しているかのように感じる。
8. まるで，それは私の所有物であるかのように感じる。
9. まるで，私はそれに対して高い所有権を有しているかのように感じる。

Perceived control

あなたはそれをどのくらい思い通りに扱うことができると感じていますか。

Willingness to accept

他の人があなたに対していくら払えば，あなたはその大事な物を手放しますか？日本円でお答えください（半角数字）。

Willingness to pay

仮にその大事な物を失ってしまったとします。買い戻せるとしたら，いくら支払いたいと思いますか？ご自身の中で最も高値と思う金額を日本円でお答えください（半角数字）。

Market price

あなたが思い浮かべた物は，一般的にいくらで売られていますか？市場価格をできるだけ正確に日本円でお答えください（半角数字）。

Self-extension tendency

1. 私は，自分のお気に入りの持ち物と特別なつながりがある。
2. 私は，自分のお気に入りの持ち物を自分の一部だと思っている。
3. 私はよく自分にとって特別な持ち物と自分との間に，個人的なつながりがあると感じる。
4. 私の一部は，人生において特別な持ち物によって定義される。
5. 私は，自分が最も好きな持ち物と親密で個人的なつながりがあるかのように感じる。
6. 私は，人生において重要な持ち物を見極めることができる。
7. 自分にとって特別な持ち物と，自分自身をどう見るかということとの間には，関連がある。
8. 自分のお気に入りの持ち物は，私という人間を示すのに重要だ。

Need for Touch

1. 店内を見て回るとき，あらゆる商品に触れる方である。
2. 商品に触れることは楽しい。
3. 店内を見て回るとき，あらゆる商品を手にとってみることは重要である。
4. 買うつもりがない場合でも，とりあえず商品に触れることが好きだ。
5. 店内を見て回るとき，いろいろな商品に触れることが好きだ。
6. 私は，店内でいろいろな商品に触れていると思う。
